# Unmasking the psychological impact of the early COVID-19 pandemic in young adults: results from a cross-sectional online survey

**DOI:** 10.3389/fpsyt.2025.1521395

**Published:** 2025-03-06

**Authors:** Omar Shazley, Michelle Teresa Wiciak, Daphne Santhosh

**Affiliations:** Department of Microbiology, Saint James School of Medicine, St. Vincent and the Grenadines, Park Ridge, IL, United States

**Keywords:** coronavirus, COVID-19, pandemic, mental health, depression, anxiety, PTSD, young adults

## Abstract

**Background:**

The COVID-19 pandemic emerged as an international public health emergency and threat to individual psychological resilience.

**Objective:**

To examine the prevalence of psychological issues and identify key associations with mental health indicators in young adults (ages 18-28) worldwide during the initial phase of the pandemic.

**Methods:**

Through a cross-sectional online survey utilizing convenience sampling, we collected data on demographics, COVID-19-related questions, depression (Patient Health Questionnaire-9), anxiety (Generalized Anxiety Disorder-7), stress/trauma (Impact of Event Scale-Revised), and fear of COVID-19 (Fear of COVID-19 scale) between September 2020-January 2021. A total of 183 were eligible analysis. All statistical analyses were set at alpha = 0.05.

**Results:**

Over 70% of participants reported mild anxiety (n=129), 80% mild depression (n=136), 40% pandemic-related trauma (n=61), and 50% high fear (n=88). Female respondents reported higher anxiety (t(173)=-3.352, <.001), depression (t(166)=-3.310, P=.001), and trauma from COVID-19 ((t(151)=-2.004, P=.047). Hispanic/Latino/a/x participants reported higher depression (F(2,156)=7.761, P<.001) and trauma scores (F(2,143)=3.999, P=.020). Age in 2020 was associated with trauma total scores (F(1,154)=4.230, P=0.041, R2 = 0.027). Individuals who were mandated a quarantine were linked to lower levels of anxiety (F(2,175)=3.442, P=.034) and depression (F(2,170)=3.092, P =.048) than those not mandated. Those quarantined with close contacts were linked to lower anxiety (t(162.184)=2.705, P =.008) and trauma (t(149)=2.169; P=.032). Close contacts’ hospitalization from COVID-19 infections were linked to lower anxiety (t(127)=2.855, P=.005), depression(t(123)=3.111, P=.002), and trauma (t(152)=-1.975, P=.050).

**Conclusion:**

The findings highlight the significant effect COVID-19 had on mental health in young adults worldwide.

## Introduction

The coronavirus disease (COVID-19) pandemic negatively changed the general population, both physically and mentally. Widespread societal disruptions (i.e., through mandated quarantines, stay-at-home measures) and socioeconomic distress exacerbated mental health struggles across various populations, especially in young adults (1–3). Initially, increased mental health burden was attributed to quarantine, but this may depend on individual and environmental factors (4). These adverse effects are not novel to COVID-19. A 2004 study after the SARS outbreak found similar adverse psychological effects associated with quarantine (5). 28.9% of respondents exhibited post-traumatic stress disorder (PTSD) symptoms and 31.2% displayed depressive symptoms, with extended quarantine periods correlated with PTSD and direct exposure to SARS patients linked to PTSD and depression (5).

Young adults (aged 18-28) are particularly vulnerable to mental health issues during COVID-19. Previous studies indicate poorer mental health outcomes compared to other age groups (6). This population encountered social disruption, isolation, financial instability, and other transitions (1, 7). They were more likely to feel lonely due to quarantine and social isolation (8). Despite evidence linking social isolation to health problems, research indicates complexity regarding mental health during the pandemic. Some research suggest young adults were less likely to seek mental health treatment, possibly contributing to more negative mental health outcomes (9–11).

Although robust national data on demographic differences in mental health exists, it often focuses on all ages rather than specific groups. Additional exploration is needed regarding race, ethnicity, and regional locations, especially given increased disparities during COVID-19. There is limited research available on how a young person’s social circle impacts psychological health, particularly when someone close has COVID-19 or is hospitalized. This variable deserves analysis because isolation and social support have affected this population. Many studies focused on pandemic-related mental health used retrospective data collection, subject to bias (12). Collecting mental health data during the pandemic is crucial to minimize this bias and provide perspective. Various pandemic timepoints associated with different quarantine measures could distort findings if ignored. Considering temporal measures can provide additional context and insight into quarantine’s impact on mental health.

This study’s research objective aims to examine the prevalence of psychological issues (depression, anxiety, fear of COVID-19, and stress and trauma from the pandemic) in young adults (ages 18-28) during the early stages of the COVID-19 pandemic (September 2020 to January 2021), when quarantine and isolation measures were stricter. Furthermore, it aims to evaluate any associations between demographic and COVID-19 variables to examine for social determinants of health (SDOH) and impact of quarantine. While exploratory in nature, we anticipated finding severely impacted mental health on all domains, with variations based on demographics, quarantine status, and social circle exposure to COVID-19.

## Methods

### Participants and procedure

A cross-sectional, observational study was conducted online using the SurveyMonkey platform (SurveyMonkey, San Mateo, CA). Participants aged 18-28 were recruited through convenience sampling via survey recruitment platforms (i.e., SurveyCircle, SurveySwap) (61.8% of participants), school-wide emails to recruit students from the medical institution (6.4% of participants), social media platforms (i.e., Meta, LinkedIn, and X) (27.4% of participants), and word of mouth (4.5% of participants). The self-administered survey took approximately 45 minutes to complete, as respondents were not compensated for participating in the survey. The anonymity of the responses was ensured.

Data were collected from September 2020 to January 2021, ensuring participants were adequately exposed to social distancing and quarantine. This timing aimed to measure psychological impacts accurately during a typical week during the pandemic. 294 respondents completed the questionnaire. Inclusion criteria required participants to be between 18-28 years old, able to consent, and correctly answer three validation questions correctly. Exclusion criteria included being outside the target age range, unable to consent, or failing to answer the validation questions correctly. 35 individuals did not meet the age range criteria, and 76 failed to answer validation questions correctly. In total, 183 responses were validated by the inclusion/exclusion criteria and were eligible for data analysis.

### Questionnaire

We employed a self-administered questionnaire that consisted of three parts. The first portion collected sociodemographic information, including gender, age (in 2020), ethnicity (responses for Hispanic/Latino/a/x, not Hispanic/Latino/a/x, and other (i.e., Romani) were collected), race, student status, employment status, income, and country of residence. Unemployed students were instructed to select “unemployed, not looking for work.” The second portion asked COVID-19-related questions about testing, infection, hospitalization, and quarantine status. It also inquired about close contacts’ experiences with COVID-19 diagnosis, hospitalization, mandated quarantine, and death. The third portion included four psychological and mental health measurement scales.

#### Mental health (depression, anxiety, psychological impact of COVID-19, Fear of COVID-19)

The Patient Health Questionnaire (PHQ-9) assessed depressive symptoms using a 4-point Likert scale for nine DSM-IV criteria (13). Score range 0-27, with from 0 (not at all), 1 (several days), 2 (more than half the days), and 3 (nearly every day), with the total sum ranging from 0 to 27. Scores range from 0-27, with depression severity categorized as minimal (0-4), mild (5-9), moderate (10-14), moderately severe (15-19), and severe (20-27). Total scores ≥ 10 indicate possible depression (14).

Anxiety was evaluated using the Generalized Anxiety Disorder 7-item (GAD-7), employing a 4-point Likert scale from 0 (not at all) to 3 (nearly every day), where anxiety levels are classified as minimum (0-4), mild (5-9), moderate (10-14), and severe (15-21) (15, 16).

The psychological impact of COVID-19 was assessed using the Impact of Events Scale-Revised (IES-R). This 22-item measure indicates potential PTSD with scores ≥ 24 (17). Assessment for COVID-19 distress evaluates intrusion (7), avoidance (8), and hyperarousal (7) on 5-point scale (18). The level of difficulty for each item experienced is scaled as “not at all” (0), “a little bit” (1), “moderately” (2), “quite a bit” (3), and “extremely” (4). Scores may indicate partial symptoms (24-32), probable PTSD (33 to 38), or severe immune system suppression (≥39) (19, 20). The mean average for every subset (i.e., intrusion, avoidance, hyperarousal) was computed in the data analysis.

The Fear of COVID-19 Scale (FCV-19S) measured stress and fear related to the virus. It uses a 5-point Likert scale that ranges from 1 (strongly disagree) to 5 (strongly agree) for total scores ranging from 7-35 (21). Higher FCV-19S scores indicate greater the fear, and results were categorized “low” or “high” based on the cut-off score (22).

### Statistical analysis

Statistical analysis was performed using SPSS version 25.0 (IBM, Armonk, NY, USA). Responses coded as “don’t know/unsure” and “refuse to answer” were coded as missing data, and were excluded from data analysis sets. Participants with incomplete responses, including “don’t know/unsure” and “refuse to answer” to validated scales, were excluded from analysis for that variable. Dependent variables included GAD-7, PHQ-9, and IES-R scores and levels. Independent variables encompassed demographics, COVID-19 testing and infection status, quarantine status, whether someone close to participant tested for COVID-19, COVID-19 impact on close contacts. Descriptive statistics were reported as mean ± standard deviation and frequency (percentage). Statistical tests included chi-square analyses, Fisher’s exact tests, paired student t-tests, ANOVA, Pearson correlations, and a linear regression model. Alpha was set at 0.05.

### Ethics approval

The study was approved by the Institutional Research Committee (IRC) at Saint James School of Medicine (research project #119), adhering to the ethical standards outlined in the 1964 Helsinki Declaration and its later amendments or similar ethical guidelines involving human participants. This study was deemed a minimal risk, with potential mental distress from answering questions. The survey included links to mental health support in case of distress. Participants were not compensated for their time. Before participating, respondents provided electronic consent. They had the right to refuse to participation, withdraw from the study, or skip questions they felt uncomfortable answering.

## Results

### Participant and COVID-19 characteristics

**Table 1** displays the general demographics studied. The sample consisted of young adults with an average of 23.43 years (SD=2.54). Most participants were female (71.1%, n=128), with 28.9% (n=52) male. Ethnically, respondents were predominantly White (79.8%, n=142), followed by Asian (14.0%, n=25), Black or African American (1.7%, n=3), and “Other” 4.5% (n=8). The majority were based in Europe (67.8%, n=124), with smaller proportions from continents including North America (23%, n=42), Asia (6.0%, n=11), Africa (2.2%, n=4), and Australia (1.1%, n=2). 106 (58.2%) were unemployed and not seeking work, 47 (25.8%) worked part-time, 24 (13.2%) worked full-time, and 5 (2.7%) reported other. 162 (89%) of participants were students.

**Table 1:** Summary of sociodemographic characteristics from eligible participants who completed the cross-sectional survey from September 2020 to January 2021 (N=183).

Variables		n (%), mean (SD)
**Age in 2020, mean (SD)**		23.43(2.54)
Gender, n (%)		
	Male	52 (28.9)
	Female	128 (71.1)
Ethnicity, n (%)		
	Hispanic or Latino/a/x	14 (8.3)
	Not Hispanic or Latino/a/x	124 (73.8)
	Other ethnicity	30 (17.9)
Race, n (%)		
	White	142 (79.8)
	Asian	25 (14.0)
	Black or African American	3 (1.7)
	Other	8 (4.5)
Continent of residence, n (%)		
	Europe	124 (67.8)
	North America	42 (23)
	Asia	11 (6.0)
	Africa	4 (2.2)
	Australia	2 (1.1)
Employment status, n (%)		
	Full-time	24 (13.2)
	Part-time	47 (25.8)
	Unemployed, not looking for work	106 (58.2)
	Other	5 (2.7)
**Student, n (%)**		162 (89.0)

Bold values highlight the largest demographic groups within each variable category, emphasizing the predominant characteristics of the survey participants.

### COVID-19 variables

**Table 2** summarizes COVID-19-related variables among the 183 participants. 40.4% were tested for COVID-19, with 6.7% positive. For quarantine status, 9.3% were currently quarantining, 33.9% had quarantined previously, and 56.8% never quarantined. Average quarantine duration was 15.18 days (SD=23.50) for current quarantines and 20.07 days (SD=20.87) for past quarantines. 38.6% reported someone close mandated to quarantine, and 30.4% had a close contact working on the front lines. 10.6% had someone close hospitalized due to COVID-19. These findings provide insights into participants’ personal experiences and indirect exposure to COVID-19 impacts.

**Table 2:** Summary of COVID-19 variables from eligible participants who completed the cross-sectional survey from September 2020 to January 2021 (N=183).

Variables		n (%), mean (SD)
**Tested for COVID-19, n (%)**		74 (40.4)
**Diagnosed with COVID-19, n (%)**		12 (6.7)
Quarantined, n (%)		
	Currently	17 (9.3)
	Past	62 (33.9)
	Never	104 (56.8)
Time spent in quarantine, mean (SD)		
	Current	15.18 (23.50)
	Past	20.07 (20.87)
**Someone close to participant mandated quarantine, n (%)**		68 (38.6)
**Someone close to participant worked on front lines during pandemic, n (%)**		55 (30.4)
**Someone close to participant hospitalized from COVID-19-related infection, n (%)**		14 (10.6)

Bold values denote the most significant data points related to COVID-19 experiences among the participants, such as the highest percentages of testing and quarantine.

### Anxiety

**Figure 1:**
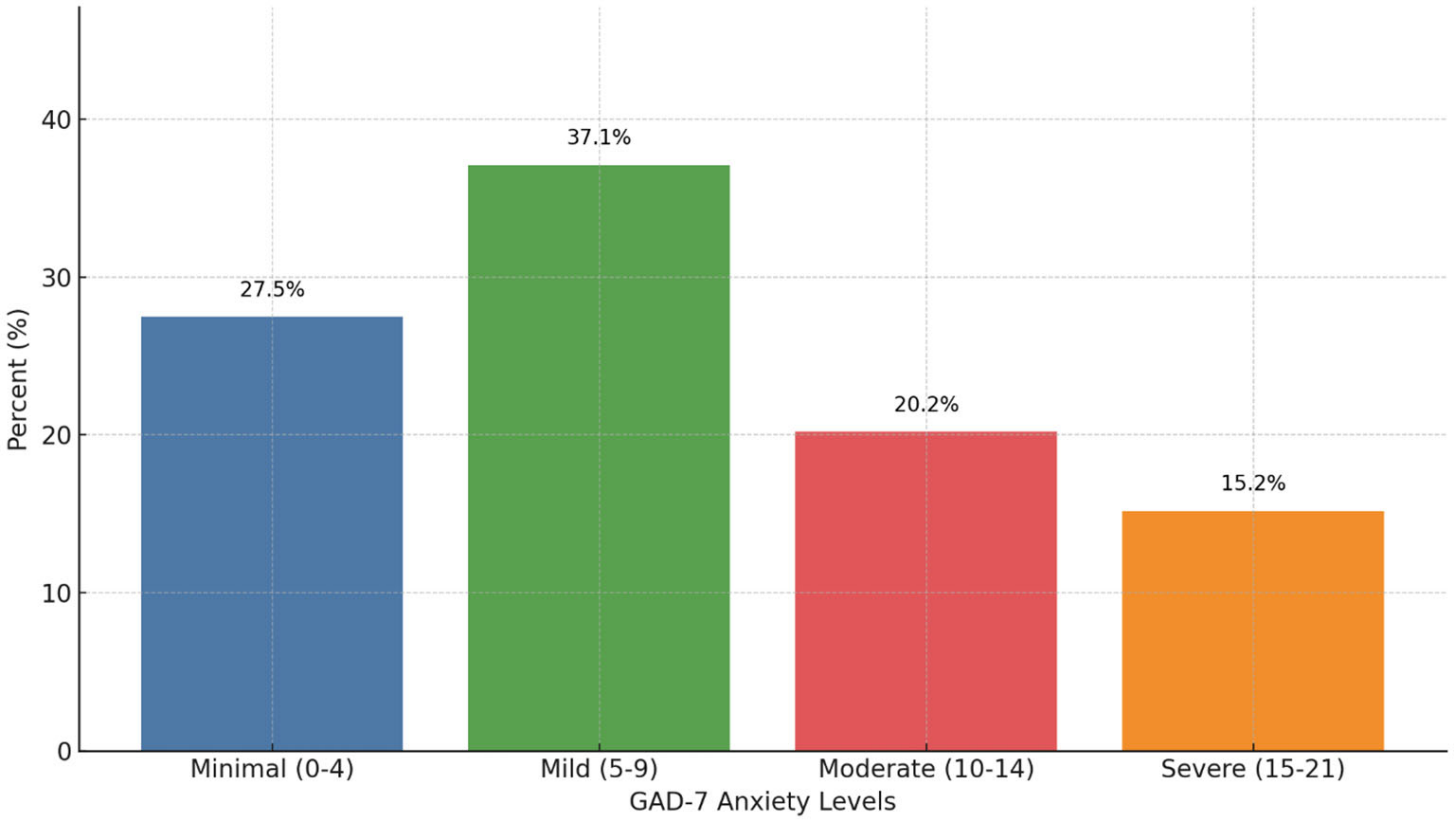
Generalized Anxiety Disorder 7-item (GAD-7) score categorical breakdown during the COVID-19 pandemic (N=178). Scores range from 0-21. 27.5% (n=49) had minimal anxiety (scores 0-4), 37.1% (n=66) had mild anxiety (scores 5-9), 20.2% (n=36) had moderate anxiety (scores 10-14), and 15.2% (n=27) had severe anxiety (scores 15-21).

The average GAD-7 score during COVID-19 was 8.19 (n=178, sd=5.46), with over 70% of participants (n=129) having at least mild anxiety. **Figure 1** shows the breakdown of GAD-7 categorical scores.

Significant differences in GAD-7 scores were found for gender (t(173)=-3.352, *P*<.001), with females scoring higher (x=9.08, SD 5.08, n=125) than males (n=6.10, SD 5.86, n=50). Quarantine status showed significant differences (F(2,175)=3.442, *P*=.034) between those never quarantined (x=7.48, SD 5.35, n=101) and those who quarantined in the past (x=9.67, SD 5.43, n=60).

Anxiety scores different significantly between those with (x=6.79, SD 4.62, n=67) and without (x=8.97, SD 5.87, n=104) close contacts mandated to quarantine (t(162.184)=2.705, *P*=.008). Similarly, scores differed for those with infection (x=4.43, SD 3.80, n=14) and without (x=8.39, SD 5.01, n=115) close contacts hospitalized due to COVID-19 (t(127)=2.855, *P*=.005).

Associations were found between anxiety levels and gender (*P*<.001), race (*P*=.010), continent (*P*<.001), quarantine status (*P*=.037), and close contact quarantine (*P*=.036) (**Table 3**).

**Table 3:** Associations between demographics and level of anxiety (from the Generalized Anxiety Disorder 7-item questionnaire) in young adults (ages 18-28) during the first wave of the COVID-19 pandemic (prior to January 2021) (N=183).

	Minimal n (%)	Mild n (%)	Moderate n (%)	Severe n (%)	Test statistic	*P*-value
Gender						
Male	25(50.0)	14 (28.0)	5 (10.0)	6 (12.0)	X2(3,175)=18.611	*P*<.001
Female	23 (18.4)	51 (40.8)	30 (24.0)	21 (16.8)		
Race						
White	40 (28.8)	58 (41.7)	23 (16.5)	18 (12.9)	Fisher’s exact=20.268	*P*=.010
Asian	6 (24.0)	4 (16.0)	9 (36.0)	6 (24.0)		
Black/African American	*	*	*	*		
American Indian	*	*	*	*		
Other	*	*	*	*		
Continent of location						
Europe	32 (26.4)	52 (43.0)	28 (23.1)	9 (7.4)	Fisher’s exact=28.124	*P*<.001
North America	10 (25.0)	11 (27.5)	4 (10.0)	15 (37.5)		
Asia	4 (36.4)	2 (18.2)	2 (18.2)	3 (27.3)		
Africa	*	*	*	*		
Australia	*	*	*	*		
Quarantine status						
Never	34 (33.7)	33 (32.7)	21 (20.8)	13 (12.9)	Fisher’s exact=12.933	*P*=.037
Currently quarantining	5 (29.4)	10 (58.8)	0 (0.0)	2 (11.8)		
Quarantined in the past	10 (16.7)	23 (38.3)	15 (25.0)	12 (20.0)		
Someone close to participant mandated quarantine						
No	26 (25.0)	34 (32.7)	22 (21.2)	22 (21.2)	X2(3,171)	*P*=.036
Yes	23 (34.3)	8 (41.9)	12 (17.9)	4 (6.0)		

*Denotes suppressed data since group has n<5.

### Depression

**Figure 2:**
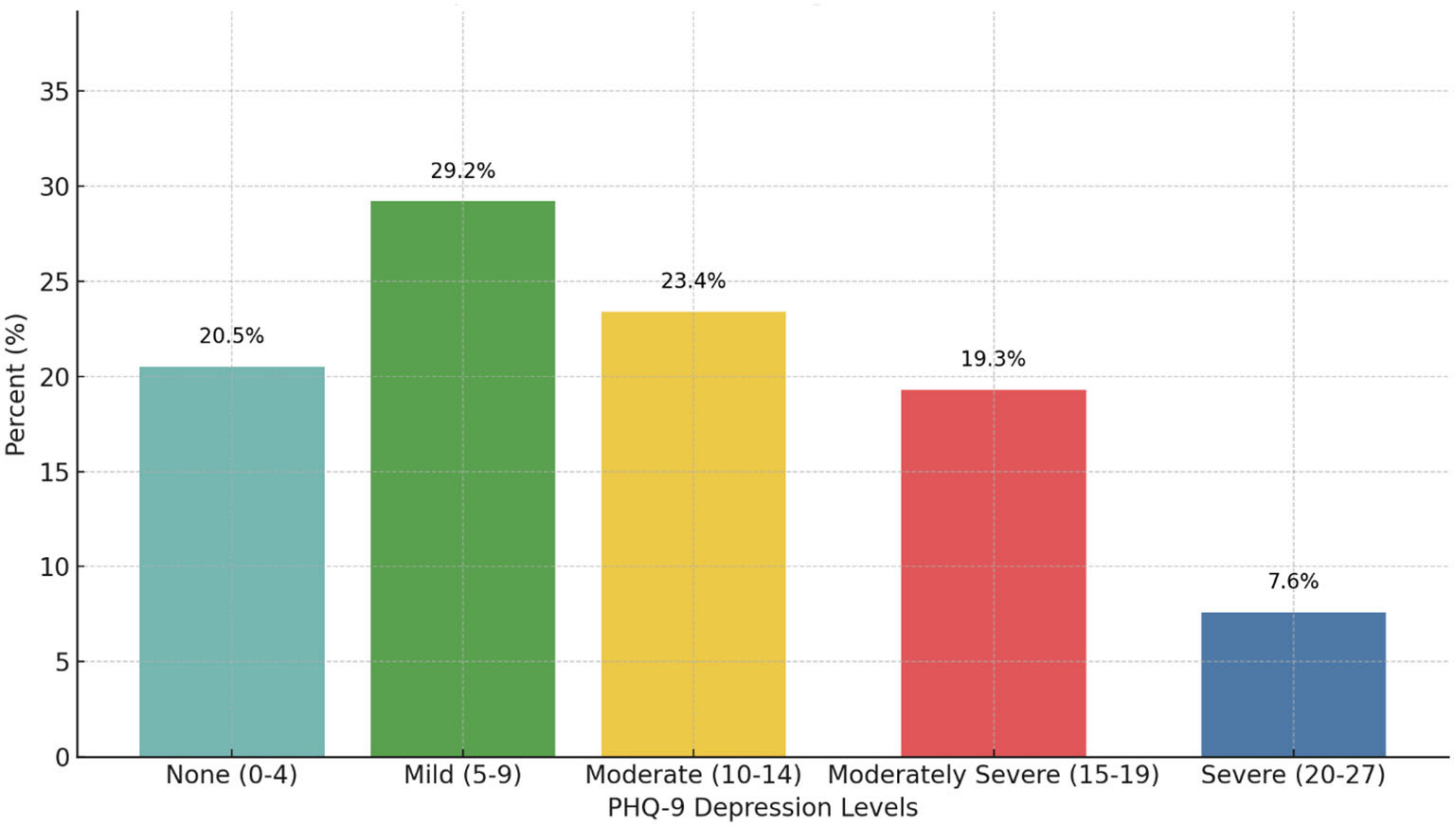
Patient Health Questionnaire 9-item (PHQ-9) score categorical breakdown during the COVID-19 pandemic (N=171). Scores range from 0-27. 20.5% (n=35) had no depression (scores 0-4), 29.2% (n=50) had mild depression (scores 5-9), 23.4% (n=40) had moderate depression (scores 10-14), 19.3% (n=33) had moderately severe depression (scores 15-19), and 7.6% (n=13) had severe depression (scores 20-27).

The average PHQ-9 score during COVID-19 was 10.14 (n=171, SD 6.12), with nearly 80% of participants (n=136) had at least mild depression. **Figure 2** depicts the PHQ-9 categorical score breakdown.

Significant differences between PHQ-9 scores were found for ethnicity (F(2,156)=7.761, *P*<.001), with Hispanic/Latino/a/x participants scoring higher (x=16.45, SD 5.70, n=11) than non-Hispanic/Latino/a/x (x=9.42, SD 5.65, n=119) and other ethnicities (x=10.90, SD 6.28, n=29). Gender difference were also significant (t(166)=-3.310, *P*=.001), with females scoring higher (x=11.12, SD 5.74, n=121) than males (x=7.74, SD 6.39, n=47).

Quarantine status showed significant differences in depression scores (F(2,170)=3.092, *P*=.048): currently quarantining (x=8.23, SD 6.51, n=17), never quarantined (x=9.54, SD 5.76, n=94), and past quarantine (x=11.62, SD 6.35, n=60).

Depression scores differed significantly (t(123)=3.111, *P*=.002) between those with (x=5.36, SD 4.20, n=14) and without (x=10.28, SD 5.72, n=111) close contacts hospitalized due to COVID-19.

Associations were found between depression levels and gender (*P*<.001), household income (*P*=.006), and student status (*P*=.048) (**Table 4**).

**Table 4:** Associations between demographics and level of depression (from the Patient Health Questionnaire 9-item questionnaire) in young adults (ages 18-28) during the first wave of the COVID-19 pandemic (prior to January 2021) (N=183).

	None n (%)	Mild n (%)	Moderate n (%)	Moderately severe n (%)	Severe n (%)	Test statistic	*P*-value
Gender							
Male	20 (42.6)	10 (21.3)	9 (19.1)	5 (10.6)	3 (6.4)	X^2^(4,168)=20.622	*P*<.001
Female	14 (11.6)	39 (32.2)	31 (25.6)	27 (22.3)	10 (8.3)		
Household income							
Less than 50k	17 (18.7)	37 (40.7)	13 (14.3)	15 (16.5)	9 (9.9)	Fisher’s exact=23.094	*P*=.006
50k-100k	7 (36.8)	1 (5.3)	8 (42.1)	2 (10.5)	1 (5.3)		
100k-150k	2 (25.0)	3 (37.5)	0 (0.0)	3 (37.5)	0 (0.0)		
150k +	2 (28.6)	1 (14.3)	3 (42.9)	0 (0.0)	1 (14.3)		
Student status							
No	9 (45.0)	4 (20.0)	4 (20.0)	1 (5.0)	2 (10.0)	Fisher’s exact=8.945	*P*=.048
Yes	26 (17.2)	46 (30.5)	36 (23.8)	32 (21.2)	11 (7.3)		

### Trauma from the pandemic

**Figure 3:**
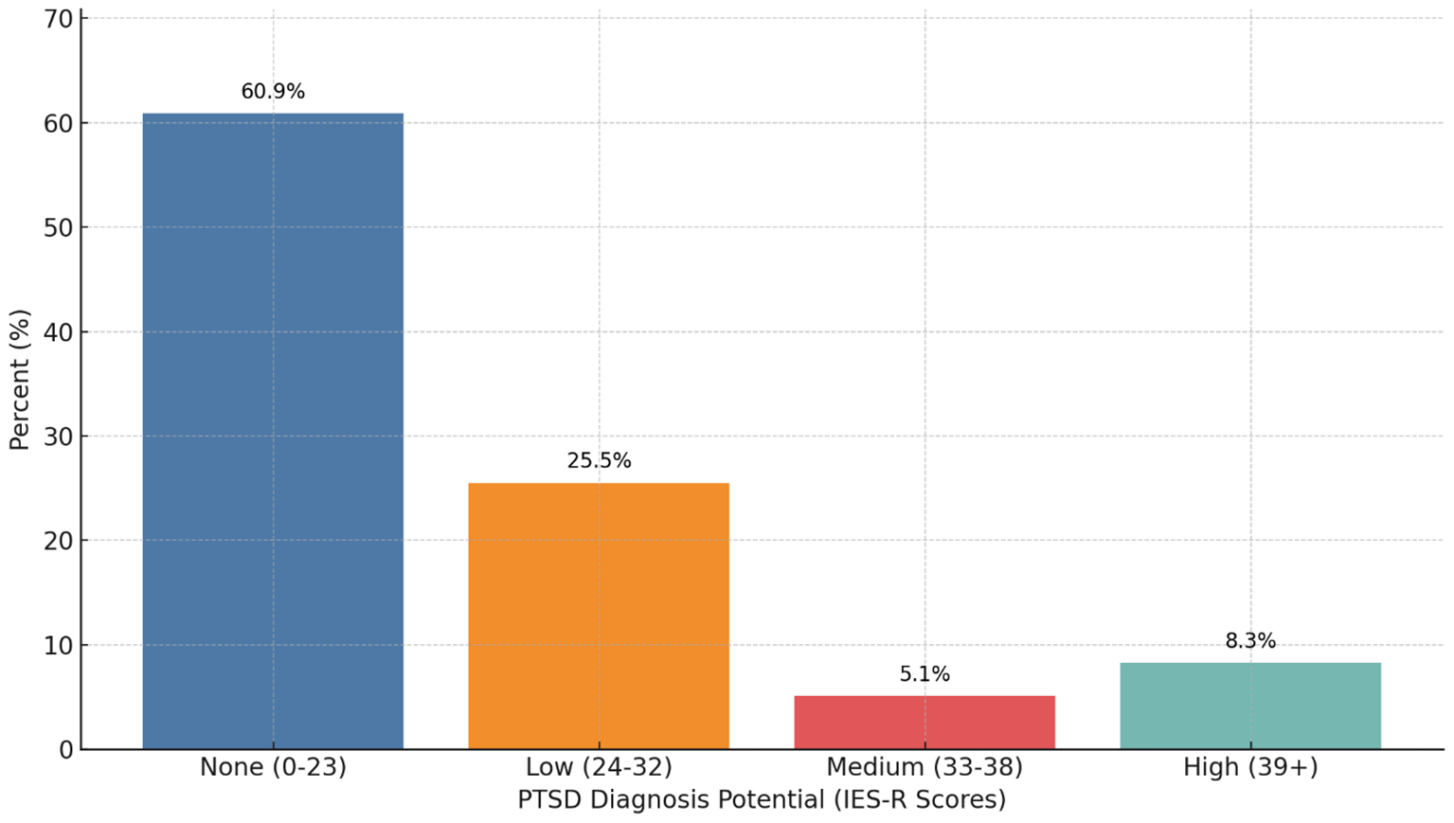
Potential for meeting criteria for post-traumatic stress disorder (PTSD) from the impact of event-revised (IES-R) scores during the COVID-19 pandemic (N=183). 60.9% (n=95) had no potential of having PTSD (scores 0-23), 25.5% (n=40) had low potential of having PTSD (scores 24-32), 5.1% (n=8) had medium potential of having PTSD (scores 33-38), and 8.3% (n=13) had high potential of having PTSD (scores 39+).

The average IES-R score during the pandemic was 19.73 (n=156, SD 14.44), with mean subscores of intrusion 0.81 (SD 0.70, n=169), avoidance 0.97 (SD 0.83, n=167), and hyperarousal of 1.01 (SD 0.76, n=179). Almost 40% of participants (n=61) had at least a low potential of meeting PTSD criteria. **Figure 3** shows the potential for PTSD based on stress scores.

Age in 2020 was associated with IES-R total scores (n=156, r= -.164, *P*=.041). Linear regression (F(1, 154) = 4.230, *P*=0.041, R^2 = 0.027, adjusted R^2 = 0.20) indicated that each year increase in age decreased IES total scores by 0.028 points (B=-0.028, 95% CI [-0.055, -0.001]).

Significant differences in IES-R scores were found for ethnicity (F(2,143)=3.999, *P*=.020), with Hispanic/Latino/a/x scoring higher (x=32.56, SD 23.02, n=9) than non-Hispanic/Latino/a/x (x=19.24, SD 13.87, n=110) and other ethnicities (x=18.15, SD 10.57, n=27). Gender differences were also significant (t(151)= -2.004, *P*=.047), with females scoring higher (x=21.24 SD 14.04, n=112) than males (x=15.98, SD 15.35, n=41).

Employment status showed significant differences (F(3,151)=3.250; *P*=.024), with full-time employed participants (x=11.24, SD 10.53, n=21) scoring lower than part-time (x=21.85, SD 16.13, n=39) or unemployed (x=21.14, SD 13.88, n=92).

Participants with close contacts mandated to quarantine had lower stress scores quarantine (x=16.29, SD 11.83, n=59) than those without (x=21.40, SD 15.43, n=92) (t(149)=2.169; *P*=.032). Similarly, those with close contacts hospitalized due to COVID-19 had lower stress scores (x=14.42, SD 12.10, n=12) than those without (x=20.10, SD 14.45, n=102) (t(152)=-1.975, *P*=.050).

Associations were found between potential PTSD and ethnicity (*P*=.031) and student status (*P*=.036) (**Table 5**).

**Table 5:** Associations between demographics and the potential of post-traumatic stress disorder (PTSD) based off Impact of Event-Revised (IES-R) scores in young adults (ages 18-28) during the first wave of the COVID-19 pandemic (prior to January 2021) in young adults during the COVID-19 pandemic (N=183).

	None n (%)	Low n (%)	Medium n (%)	High n (%)	Test statistic	*P*-value
Ethnicity						
Not Hispanic or Latino/a/x	70 (63.6)	25 (22.7)	5 (4.5)	10 (9.1)	Fisher’s exact=12.291	*P*=.031
Hispanic or Latino/a/x	3 (33.3)	2 (22.2)	2 (22.2)	2 (22.2)		
Another ethnicity	15 (55.6)	11 (40.7)	1 (3.7)	0 (0.0)		
Student status						
No	13 (86.7)	0 (0.0)	1 (6.7)	1 (6.7)	Fisher’s exact=7.498	*P*=.036
Yes	81 (57.9)	40 (28.6)	7 (5.0)	12 (8.6)		

### Fear of COVID-19

The average fear score of COVID-19 was 16.27 (SD 5.70; n=182), with 48.4% (n=88) of participants showing high fear levels. A significant difference in fear scores (t(173)=2.327; *P*=.021) was found between those with close contacts mandated to quarantine (x=15.01, SD 5.87, n=68) and those without (x=17.06, SD 5.52, n=107). This suggests that having someone close undergo quarantine was associated with lower fear levels.

## Discussion

This study examined the prevalence of psychological impact during the early COVID-19 in young adults and the associations mental health factors had with SDOH. Our findings revealed a high prevalence of mild anxiety (>70%), depression (80%), pandemic-related PTSD (~40%), and fear of COVID-19 (50%). These findings are consistent with current literature, where young adults experience significant mental health challenges (6, 7, 9, 23–25). It has even been found that those without a pre-disposing anxiety or depressive condition prior to the pandemic were even significantly impacted, with evidence to suggest that those without mental health conditions had a greater deterioration than those with a pre-existing condition (23). These findings emphasize the reach of the COVID-19 pandemic had on the young adult population, with the increased need for mental health support, as well as understanding between groups of differing mental health outcomes.

The average GAD-7 score of 8.19 in our sample indicated mild anxiety, aligning with previous research (23–25). Similar to existing literature, young female adults exhibited higher susceptibility to anxiety during the pandemic (24, 26). This gender discrepancy may have predated the pandemic, given historically higher anxiety rates in females (25, 27). Interestingly, individuals with a history of quarantine showed elevated anxiety scores, demonstrating the potential negative impact of isolation and loneliness on mental health (28, 29). This was observed only in those with past quarantine experiences, not those currently quarantining, suggesting that the timing of quarantine may influence anxiety levels. There were higher rates of moderate to severe anxiety among Asian participants than Whites, which could be because of increased racism towards Asians during COVID-19 (30). Our study notably revealed an unexpected relationship between a young adults’ social circle and anxiety levels. Participants with close contacts who experienced quarantine or hospitalization reported lower anxiety levels. This counterintuitive finding contrasts with previous observations that pandemic-related anxiety often stemmed from concerns about COVID-19 diagnoses within one’s social circle (31, 32). Possible explanations include a better understanding of the disease through first-hand experience or reduced feelings of isolation due to shared quarantine experiences.

The average PHQ-9 score was 10.14, indicating moderate depression levels. This finding aligns with existing research that suggests a general increase in depression rates, potentially influenced by factors such as academic stress and social isolation (24, 26, 33). Like depression, we observed gender differences, with females more likely than males to have severe depressive symptoms. This trend may also have predated the pandemic (24, 27). Recent evidence further suggests that females not only experience more severe depression than males but also face worse long-term COVID-19 effects, both of which could potentially contribute to increased cardiovascular disease (CVD) risk in women in the future (34). Yet, participants currently in quarantine exhibited lower depression scores compared to those who had never quarantined or had done so previously. This finding aligns with a recent study that supported higher levels of psychological distress and lower life satisfaction due to stringent quarantine measures but not necessarily increased depression or anxiety (35). While our study did not directly measure social connectedness and support, these factors are recognized as potential mediators of depression during periods of social distancing. Hou et al. (2021) explored this concept, identifying social support as a risk and protective factor for psychological distress among young adults during the pandemic (36).

A relatively novel and counterintuitive finding suggests that participants who had close contact hospitalized due to COVID-19 reported lower depression scores. This aligns with recent research suggesting that anxiety and depression levels can be lower in individuals during the pandemic, possibly due to social exposure (31). While current literature primarily focuses on depression in individuals following their own hospitalization, few studies exist on the psychological impact hospitalization has on one’s social circle. This finding lends a new perspective on the complex, bidirectional relationship between social COVID-19 exposure and mental outcomes.

Various demographic disparities regarding depression also emerged from this study. Hispanic/Latino/a/x individuals reported higher depression scores, a disparity potentially rooted in pre-existing inequalities exacerbated by increased financial strain, job insecurity, and limited healthcare access (37). Depression severity varied across income backgrounds, with some unexpected patterns emerging. Both lower-income (below $50K) and upper-middle-income ($100K-150K) groups showed higher rates of mild depression. Those households within the $100-150K range also exhibited a higher proportion of moderately severe depression cases. These findings challenge the traditional view of socioeconomic status (SES) as an SDOH during COVID-19, yet recent literature suggests that individuals with higher education levels, often associated with higher incomes, may have experienced greater declines in well-being (37, 38). Our findings also uncovered an intriguing association between student status and depression severity. Non-students were more likely to report no depression compared to their student counterparts, highlighting the complex interplay between SES, educational pursuits, and mental health. These findings signify the need for further evaluation of the role of income and other socioeconomic factors on mental health during global pandemics.

Overall, our participants experienced significant pandemic-related stress, with nearly 40% potentially meeting the criteria for PTSD symptoms, aligning with current literature (39, 40). We identified several demographic factors associated with varying stress levels. Trauma scores decreased with increasing participant age, suggesting that older individuals within our sample reported lower levels of trauma. While age-related associations with pandemic-induced trauma are not well-documented in young adults, older individuals may be less likely to develop PTSD from COVID-19 (41). This finding is corroborated by a cross-sectional survey in Mexico, where younger individuals experienced higher psychological distress during COVID-19 (42). Understanding resilience and protective factors between various ages should be a focus of research in the future, as this can lead to personalized approaches for mental health interventions for young adults. Additionally, significant differences were observed among ethnicities, particularly in the Hispanic and Latino/a/x populations. Yet, this is controversial in literature, as some individuals from diverse ethnic backgrounds report resilience and personal growth as facilitators for mental health during the pandemic that helped mitigate significant depression and anxiety levels (43). Despite limited research, existing studies suggest that these groups, along with low-income populations and women, have been disproportionately affected by pandemic-related stressors, including financial hardship, healthcare accessibility, discrimination, and immigration challenges (43, 44). Our findings also revealed higher stress levels among young females, consistent with previous studies indicating women’s increased susceptibility to severe depressive and post-traumatic symptoms, especially among those diagnosed with COVID-19 (45, 46). Lastly, employment status emerged as a significant factor, with unemployed or part-time employed individuals reporting higher trauma scores than their full-time employed counterparts. This aligns with existing literature highlighting socio-demographic characteristics as determinants of individual psychological responses to the pandemic (46). Our findings underscore the need for targeted mental health interventions and financial support for vulnerable populations, particularly women, ethnic minorities, and those with lower SES (46).

An unexpected relationship emerged between participants’ social networks and their pandemic-related distress. Contrary to previous assumptions, participants reported lower distress scores if they had close contacts who were either quarantined or hospitalized due to COVID-19. Participants exhibited reduced fear of COVID-19 when a close contact was quarantined, suggesting that proximity to those directly affected by the pandemic may have alleviated stress rather than exacerbating it. These findings challenge previous research, suggesting that prioritizing the well-being of family and friends over personal health increases the likelihood of PTSD symptoms, and individuals in quarantine, young people, and those with close contacts infected with COVID-19 are more likely to experience virus-related fear (47, 48). Various reasons can describe these discrepancies, including emotional resilience, perspective, empathy, and shared bonding experiences. Future research should be conducted to understand this relationship better, as it can be a mediator in facilitating pandemic-related stressors.

### Implications and future directions

This work highlights the need to continue understanding mental health issues in young adults during worldwide events, particularly pandemics, as well as focus on exploring SDOH in this population. Utilizing the socioecological theory could be used to place perspective on all the influencing factors of the young adult population, especially when considering the impact of various interpersonal factors (like age, gender, student status, and income), social circles, quarantine status, and other systemic factors (49). A better understanding of the influence of social networks on mental health during the pandemic is a required future direction because of this work, as the concept of “social network” is vague and encompasses all close people to a participant in this study. It would be beneficial to understand the different social networks (i.e., friends, family, neighbors, teachers, coworkers) and their impact on an individual, especially since this study showed the significant impact of social circles on anxiety and depressive symptoms in young adults during the pandemic. This can then be utilized to create robust interventions utilizing the various levels in SEM to support an individual’s mental health during a pandemic in an evidence-based manner.

### Limitations

The methodology of the online study design and self-reported measures may have introduced bias. The use of convenience sampling and self-reported measures reduces the generalizability of the findings; the sample population was skewed towards female, white/Caucasian students from North America and Europe. Furthermore, the smaller sample size limited country-specific analysis, making it difficult to apply mandated public health interventions by group. This study did not collect information on pre-existing psychiatric disorders and has limited variables for social connection. While associations between COVID-19 factors and mental health outcomes were observed, these cannot confirm direct cause-and-effect associations. The exploratory nature of this study limits its ability to inform intervention design, as it focuses on identifying patterns rather than establishing causality.

## Conclusion

This study reinforces existing literature and provides new insights into the COVID-19 pandemic’s impact on young adults’ mental health. We found high prevalence of depression, anxiety, pandemic-related distress, and fear. Factors such as age, gender, ethnicity, and income significantly influenced mental health outcomes. Social circles played a crucial role, particularly when close contacts were quarantined or hospitalized due to COVID-19. These findings highlight the complexity between social factors and mental health during the pandemic. To improve global mental health, it is essential to incorporate biopsychosocial approaches in clinical care, continuously monitor mental health outcomes, and identify risk factors among young adults. This approach could lead to more effective interventions and support strategies.

## Data Availability

The original contributions presented in the study are included in the article/supplementary material. Further inquiries can be directed to the corresponding author.
